# Evolutionary consequences of antibiotic use for the resistome, mobilome and microbial pangenome

**DOI:** 10.3389/fmicb.2013.00004

**Published:** 2013-01-22

**Authors:** Michael R. Gillings

**Affiliations:** Department of Biological Sciences, Macquarie UniversitySydney, NSW, Australia

**Keywords:** metagenomics, evolvability, pollution, pangenome, resistome, parvome, mobilome

## Abstract

The widespread use and abuse of antibiotic therapy has evolutionary and ecological consequences, some of which are only just beginning to be examined. One well known consequence is the fixation of mutations and lateral gene transfer (LGT) events that confer antibiotic resistance. Sequential selection events, driven by different classes of antibiotics, have resulted in the assembly of diverse resistance determinants and mobile DNAs into novel genetic elements of ever-growing complexity and flexibility. These novel plasmids, integrons, and genomic islands have now become fixed at high frequency in diverse cell lineages by human antibiotic use. Consequently they can be regarded as xenogenetic pollutants, analogous to xenobiotic compounds, but with the critical distinction that they replicate rather than degrade when released to pollute natural environments. Antibiotics themselves must also be regarded as pollutants, since human production overwhelms natural synthesis, and a major proportion of ingested antibiotic is excreted unchanged into waste streams. Such antibiotic pollutants have non-target effects, raising the general rates of mutation, recombination, and LGT in all the microbiome, and simultaneously providing the selective force to fix such changes. This has the consequence of recruiting more genes into the resistome and mobilome, and of increasing the overlap between these two components of microbial genomes. Thus the human use and environmental release of antibiotics is having second order effects on the microbial world, because these small molecules act as drivers of bacterial evolution. Continued pollution with both xenogenetic elements and the selective agents that fix such elements in populations has potentially adverse consequences for human welfare.

## INTRODUCTION

The discovery of antibiotics and their use in the treatment of bacterial infections was one of the major scientific achievements of the 20th century. However, over the last 60 years, there has been a spectacular and rapid evolution of antibiotic resistant strains of bacteria. This has culminated in the appearance of pathogens with resistance to a wide range of antibiotics, and a rise of similarly resistant opportunistic organisms (Davies, 2007; Davies and Davies, 2010). Antibiotic resistance is a critical problem for humans and their domestic animals, and is recognized as such by workers in government, clinical practice, research, and industry (Bush et al., 2011).

The focus of research and thinking about this problem has mainly been from an anthropogenic viewpoint. However, at its heart, antibiotic resistance is an environmental and evolutionary problem. Humans are now the greatest evolutionary force on the planet (Palumbi, 2001), and it would help us understand and manage the resistance problem to investigate resistance from a broader perspective, most notably in terms of the interplay between ecology, evolutionary dynamics, and natural selection. A number of authors are now thinking about the ecology and natural history of antibiotics and resistance genes. This approach will inform clinical and veterinary practices and will improve our understanding of evolutionary processes (Aminov and Mackie, 2007; Baquero et al., 2009; Fajardo et al., 2009; Stokes and Gillings, 2011).

There is evidence that the antibiotic revolution may have second order effects, and is influencing the evolution of the entire microbial biosphere (Martinez, 2008; Baquero, 2009; Couce and Blázquez, 2009; Gillings and Stokes, 2012). This paper attempts to place antibiotics, resistance genes, their vectors, and their hosts into an evolutionary perspective. By understanding the evolutionary history of these genes and molecules, we place ourselves in a better position to predict their future (Martinez et al., 2007; Courvalin, 2008; Conway Morris, 2010).

## THE NATURAL HISTORY OF ANTIBIOTICS

The term “antibiotic” reflects our anthropocentric viewpoint. Antibiotics are not a discrete class of molecules, but rather, encompass a broad range of structural and molecular families, united by their ability to inhibit microbial growth at high concentrations. The original use of the word “antibiotic” was a generic term that simply reflected the outcome of a laboratory test (Waksman, 1973; Davies and Davies, 2010). In modern terms, an antibiotic has broadly come to mean any synthetic or naturally occurring low molecular weight molecule that inhibits bacterial growth.

It is clear that there are millions of low molecular weight compounds in natural environments, and that in high enough concentrations some of these could exhibit an antibiotic effect. However, it is unlikely that such compounds ever reach inhibitory concentrations in nature, and there is a growing realization that their primary role is not necessarily in cross-species warfare, but may largely lie in cell–cell communication (Linares et al., 2006). This change in viewpoint began with the discovery of bacterial quorum sensing, a system that allows communication between cells of the same or different species via signaling molecules called auto-inducers. Quorum sensing is found in diverse organisms, and the scale and extent of signaling systems in general has led to the revelation that the microbial world is in a state of constant and complex communication (Lyon and Muir, 2003; Henke and Bassler, 2004; Stevens et al., 2012). Consequently, many of the small molecules produced by bacteria may be involved in additional forms of communication between cells. The compounds we know as “antibiotics” are a minor subset of this world of small molecules.

The growth-inhibiting concentrations of antibiotics used in clinical practice are unlikely to ever be reached via natural synthesis, and consequently, antibiotics probably have significant effects at sub-inhibitory concentrations, without affecting bacterial growth rates. It has now been shown that sub-inhibitory concentrations of various antibiotic classes have effects on gene transcription. Up regulation and down regulation of diverse genes has been demonstrated, with some estimates suggesting that as many as 5% of gene promoters might be affected (Goh et al., 2002; Linares et al., 2006). Some antibiotic classes modulate expression of particular genes (Tsui et al., 2004). The types of functions affected are diverse, including genes for protein synthesis, carbohydrate metabolism, transport/binding proteins and genes of as yet unknown function (Goh et al., 2002; Lin et al., 2005).

The cascade of gene expression induced by sub-inhibitory concentrations of antibiotics leads to phenotypes of adaptive significance. These include flagella biosynthesis and the ability to colonize biotic and abiotic surfaces (Seshasayee et al., 2006). As might be expected, some of these phenotypes have direct relevance for species–species interactions and responses to host cells, such as virulence, biofilm formation, and motility (Goh et al., 2002; Hoffman et al., 2005; Linares et al., 2006; Marr et al., 2007; Shank and Kolter, 2009). Collectively, these observations strongly suggest that the antibiotics used in medical treatment are a small subset of the diverse secondary metabolites used in the microbial world for signaling and other cell–cell interactions. Their clinical use is based on a serendipitous ability to inhibit growth at unnaturally high concentrations. There is a growing realization that the discovery, clinical use, and evolution of antibiotics must be considered against the background of their roles in natural environments (Davies et al., 2006; Yim et al., 2007; Fajardo and Martínez, 2008; Ryan and Dow, 2008).

## A GENERIC MODEL OF SMALL MOLECULE SIGNALING

**FIGURE 1:**
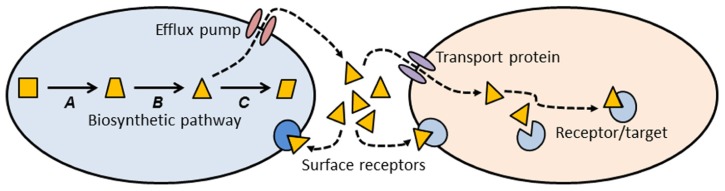
**Conceptual schematic illustrating the production, export, and target sites of a small molecule biosynthetic cluster.** The metabolic pathway for synthesis of a small molecule is encoded by genes *A*, *B*, and *C*. One of the intermediates (triangle) is exported from the cell via an efflux pump, where it can then bind to cell surface receptors, or enter a second cell via a membrane transport protein. Inside the second cell there may be binding sites on additional target molecules. Most of the molecules we know as antibiotics may be a subset of this more general class of signal-receptor systems.

Thinking about antibiotics as a subset of signaling molecules allows us to construct simple conceptual models that might establish some general principles about antibiotics and antibiotic resistance (**Figure 1**). A signaling molecule must be made by a metabolic pathway, whose enzymes are encoded by genes (*A, B, *and *C* in **Figure 1**). Any intermediate small molecule in this pathway can be exported as a signaling molecule, most probably using an efflux pump. Small molecules then diffuse into environmental space, where they can bind to receptors on the cell surface of the producing species (intra-species signaling), or to cell surface receptors of different species (inter-species signaling). Alternatively, small molecules can be imported into cells via a membrane transport protein, and then bind to a target within the cell (**Figure 1**). Binding of signaling molecules can influence transcription or biochemical pathways, and thus affect the phenotypic attributes of the receptor cell.

This conceptual model (**Figure 1**) allows us to consider antibiotics and resistance from a different and broader perspective. Antibiotics can be thought of as a subset of small biosynthetic molecules, albeit those which happen to have the property of inhibiting bacterial growth at high concentrations. Given the fact that bacteria have been evolving and interacting for some 3.8 billion years, it should not be surprising that the genes encoding biosynthetic and catabolic pathways for small molecules have an ancient evolutionary history. Pathways for synthesis of erythromycin and streptomycin date to more than 600 million years ago (Baltz, 2005), and it has been estimated that the serine β-lactamases date back 2 billion years (Hall and Barlow, 2004). Under this view “antibiotics” predate humans by some billions of years.

Note that the use of an intermediate in the biosynthetic pathway as a signaling molecule (**Figure 1**, triangle) means that the producing cell already encodes an enzyme capable of modifying the signal molecule. Under other circumstances, such a gene might confer resistance to high concentrations of the agent. Many biosynthetic gene clusters that make “antibiotics” are also known contain genes that confer “resistance” to those same antibiotics (Pootoolal et al., 2002; Huang et al., 2005). Given that gene clusters for secreted molecules are frequently subject to lateral gene transfer (LGT; Fischbach et al., 2008; Nogueira et al., 2009), it should not be surprising that there is clear evidence for lateral transfer of antibiotic resistance genes from environmental bacteria into pathogens (Gillings et al., 2008a,b; Forsberg et al., 2012).

Small molecules can exit the producing cell via a wide diversity of membrane bound efflux pumps (**Figure 1**; Paulsen et al., 1996; Desvaux et al., 2004; Poole, 2005; Bay and Turner, 2009), each of which, in turn, may be able to export diverse molecular species (Zgurskaya and Nikaido, 2000; Kuete et al., 2011). Such pumps have evolved as mechanisms to export natural substances produced internally, or those that are produced by other cells (Martinez et al., 2009b). Efflux pumps may also be essential for colonizing and persisting in eukaryotic hosts (Piddock, 2006). It is thus clear that antibiotic resistance mediated by efflux pumps is a side-effect of a more general molecular export system.

Genes for efflux pumps are commonly found in bacteria that produce antibiotics (Petković et al., 2006), and these genes are often found embedded within biosynthetic gene clusters for antibiotics, thus conferring efflux ability (and “resistance”) on the producing organism (Méndez and Salas, 2001). Again, the “resistance” gene is preexistent, and located within a gene cluster characterized by LGT (Nogueira et al., 2009).

Once exported from the cell, a signaling molecule is free to bind with receptors on the surface of neighboring cells (**Figure 1**). Point mutations in the genes for these receptors might alter the binding site and thus generate a “resistance” phenotype. Alternatively, the signaling molecule may be taken up by a membrane transport protein and imported into a new cell. A surprising diversity of bacteria make use of such transport proteins to import small molecules such as antibiotics, which they can then use as a sole carbon source (Dantas et al., 2008). Lateral transfer of the genes for such catabolic processes would confer resistance on the recipient cell. Resistance could also be generated by mutation to the gene encoding the membrane transport protein, preventing entry of the signaling molecule. Once inside a recipient cell, a signaling molecule may bind to a target site, and thus exert an effect. Mutation of the gene encoding the target site may abolish binding and prevent the effect. In the case of an antibiotic, such a change would be called a resistance mutation.

Although the model I have presented above is simplistic, nevertheless it has good explanatory power for understanding the ecology and evolution of both antibiotics and their corresponding resistance genes. Antibiotics are seen as a subset of a diverse group of small molecules whose primary function is in cell–cell interactions. Genes for synthesizing, transporting, and catabolizing these small molecules can be co-opted as resistance genes when cells are exposed to unnaturally high concentrations of these compounds. The high frequency of lateral exchange of biosynthetic gene clusters for small molecules (together with their associated efflux pumps and catabolic functions) explains the penetration of efflux pumps and degradative enzymes into species unrelated to the original “antibiotic” producer.

## ANTIBIOTICS, RESISTANCE GENES, AND THE GLOBAL MICROBIOME

**FIGURE 2:**
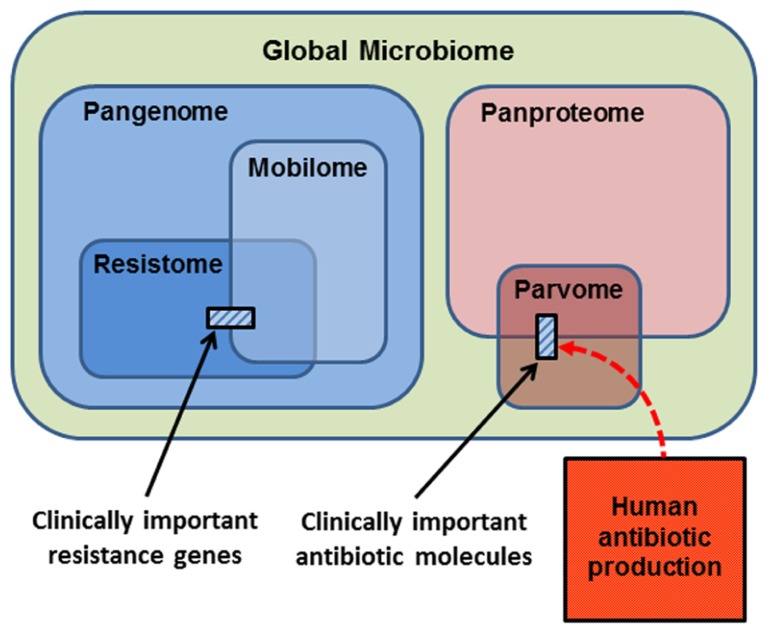
**Conceptual representation of the biological molecules of relevance to antibiotic resistance.** The small cross-hatched boxes represent the antibiotics and resistance genes of relevance to clinical practice. Respectively, these are a small subset of the world of small bioactive molecules (the parvome), and the world of potential resistance determinants (the resistome). The resistome comprises the genes that potentially encode resistance to antibiotics. The mobilome comprises the mobile proportion of bacterial genomes. The mobilome and resistome overlap, since many resistance genes are located on mobile elements. Both the resistome and mobilome are a subset of the total coding capacity of prokaryotic cells, the pangenome, which is expressed as the panproteome. Note that only a small proportion of the parvome is utilized by humans for antibiotic purposes, and that the scale of commercial antibiotic production probably overwhelms the natural production of these molecules by the entire global microbiota.

In thinking about the ecology and evolution of antibiotics and resistance genes, we can summarize the sub-components upon which selection can act in a series of Venn diagrams (**Figure 2**). The largest category is the **Global Microbiome**, encompassing all the prokaryotic cells in the biosphere. This may contain 4–6 × 10^30^ cells and hold a significant proportion of the carbon, nitrogen, and phosphorus found in living things (Whitman et al., 1998). The phenotypes exhibited by the global microbiome are encoded by the microbial **Pangenome**, that is, the set of genes present in all the genomes of all the prokaryotes in the biosphere (Medini et al., 2005; Tettelin et al., 2008; Lapierre and Gogarten, 2009). Estimates of the composition of the pangenome have been made, based on the rapid accumulation of bacterial genome and metagenome sequences. Some 250 gene families are common to all bacterial genomes (the extended core genome) and about 8,000 gene families are niche-specific genes essential for survival in particular environments (the character genome). The bulk of pangenomic diversity comprises more than 139,000 gene families that occur as accessory genes, these being found dispersed amongst single strains, serovars or species (Lapierre and Gogarten, 2009). The combined coding capacity of the pangenome is expressed as the **Panproteome**, this being the sum of all the proteins encoded and produced by the microbial realm.****

A proportion of the pangenome is highly mobile, and able to move comparatively freely between prokaryote species in the process known as LGT. Elements that specialize in moving DNA within and between genomes include plasmids, transposons, integrons, insertion sequences, and integrative conjugative elements. These elements and the genes they carry are collectively known as the **Mobilome** (**Figure 2**; Siefert, 2009; Leplae et al., 2010). In principle, and over evolutionary timescales, any part of the pangenome can be mobilized by LGT. The mobilome helps to generate enormous diversity within prokaryote genomes by recombination between various mobile elements to create a series of complex mosaic structures (Garriss et al., 2009; Toleman and Walsh, 2011; Toussaint and Chandler, 2012).

The **Resistome** is defined as the collection of all genes that could contribute to a phenotype of antibiotic resistance (D’Costa et al., 2006; Wright, 2007, 2010; Forsberg et al., 2012). This is a purely functional definition made from a human and medical point of view, for as we have seen, the resistome encompasses diverse genes whose original functions were not simply for avoiding the effects of antibiotics. Nevertheless, it is a useful concept, because it underscores the point that resistance genes originate from environmental bacteria. A database of antibiotic resistance genes has been developed (Liu and Pop, 2009), but the 20,000 genes already listed are only a small proportion of the resistome, since investigation of soil metagenomes has revealed diverse and divergent resistance genes capable of dealing with multiple classes of antibiotic (Riesenfeld et al., 2004; Allen et al., 2008). Genes encoding antibiotic resistance are ancient components of the pangenome, since they have been recovered from 30,000 year old permafrost (D’Costa et al., 2011), and from a cave microbiome that has been isolated for 4 million years (Bhullar et al., 2012). Note that the resistome overlaps with the mobilome (**Figure 2**), because many antibiotic resistance genes are found on mobile elements, allowing the resistome to be widely disseminated by LGT (Norman et al., 2009; Fondi and Fani, 2010; Skippington and Ragan, 2011). Also note that the resistance genes and mobile elements that are of concern for clinical antibiotic resistance (cross-hatched box, left hand side **Figure 2**) are just a fraction of the resistome and mobilome.

The **Parvome** is the world of small bioactive molecules produced by cells (also sometimes called secondary metabolites). The parvome includes important classes of molecules such as polyketides, aminoglycosides, terpenoids, alkaloids, and non-ribosomal peptides, many of which have antimicrobial activity (Davies, 2011; Davies and Ryan, 2011). The parvome overlaps with the proteome, because some of these small bioactive molecules are peptides. Note however, that only a small proportion of the parvome is utilized by humans for its antimicrobial properties (cross-hatched box, right hand side **Figure 2**). Commercial production of antibiotics overwhelms their natural synthesis, such that humans are now the major source of antibiotics in the general environment (**Figure 2**; Sarmah et al., 2006; Davies and Davies, 2010).

## EVOLUTIONARY CONSEQUENCES OF THE ANTIBIOTIC AGE

Having examined the relationships between the key components of relevance to antibiotic production and resistance (**Figure 2**), we can now examine how each might be affected by human activity. There are several potential dimensions to these effects, including changes to the abundance and diversity of each component, and whether the effects are transient or permanent.

### THE PARVOME

Antibiotics are released into the environment via human waste streams because a significant proportion of prophylactic antibiotics are excreted essentially unchanged (Sarmah et al., 2006; Le-Minh et al., 2010). Although it is difficult to obtain precise estimates for commercial production of antibiotics, it probably amounts to millions of metric tons per year (Segura et al., 2009). Because of the volume of antibiotics generated by human activity, the majority of the environmental load of antibiotics now originates from this commercial production (**Figure 2**; Davies and Davies, 2010). Certainly the local concentrations of this subset of the parvome have markedly increased due to human activity. Further, the diversity of small bioactive molecules has increased, because many antibiotics are synthetic modifications of natural structures. There is now a zone of influence around all human activities where the abundance and diversity of the parvome is increased. Such zones increase selective pressures on the local microbiome, not just the intended targets of antibiotic therapy (Martinez et al., 2009a; Taylor et al., 2011), and may also interfere with cell–cell communication.

### THE RESISTOME

Increasing the concentration of antibiotics in the environment has effects on both the diversity and the abundance of genes belonging to the resistome. Selection for cells that carry resistance determinants increases their relative abundance, and thus increases the abundance of genes that confer resistance. Further, the ability of many elements of the resistome to undergo LGT, means that identical resistance genes can now be found in diverse bacterial species, from clinical contexts, from domestic animals, wild animals, and in locations apparently distant from the influence of developed societies, such as the Arctic, Antarctica, and the Amazonian jungle (Pallecchi et al., 2008; Sjolund et al., 2008; Bartoloni et al., 2009; Stokes and Gillings, 2011). That humans are responsible for this phenomenon is demonstrated by the positive correlation between the abundance of resistome elements of clinical significance and proximity to human activity (Skurnik et al., 2006; Hardwick et al., 2008; Thaller et al., 2010; Nardelli et al., 2012).

Selection pressure is particularly acute in human waste streams, where resistance genes are shed, mixed with high concentrations of antibiotics and other selective agents (Baquero et al., 2008; Schlüter et al., 2008; Moura et al., 2010). Exposure of environmental microorganisms to this mixture encourages fixation of LGT events, spreading elements of the resistome into diverse strains and species, thus further increasing their abundance, and their penetration into new hosts and niches.

Exposure to sub-inhibitory concentrations of antibiotics induces expression of error-prone DNA polymerases, increasing the basal mutation rate (Kohanski et al., 2010; Thi et al., 2011). This has the effect of generating additional genomic diversity, and of co-opting additional genes into the resistome via mutational changes to housekeeping or accessory genes whose original function was not antibiotic resistance (Dantas and Sommer, 2012). As a second order effect, constant low level antibiotic exposure selects for lineages with inherently higher rates of mutation, generating additional diversity across the entire pangenome via drift and selection (Earl and Deem, 2004; Pigliucci, 2008; Couce and Blázquez, 2009; Gillings and Stokes, 2012).

Human activity has increased the abundance of resistome elements by selection, as shown by their increase in frequency in soils collected over the last 70 years (Knapp et al., 2009), and by their lateral transfer to diverse species. We have also increased the diversity and membership of the resistome by fixation of *de novo* mutations and by the co-option of genes as resistance determinants. Thus the size of the resistome, as a proportion of the pangenome, is probably becoming larger (**Figure 2**).

### THE MOBILOME

The clinical use and environmental dissemination of antibiotics has had significant effects on the abundance and diversity of elements within the mobilome. Selection for antibiotic resistance has fixed lineages carrying diverse resistance determinants on a similarly diverse array of mobile elements. Such complex mobile elements now occur at high frequency in human-dominated systems, and in more natural ecosystems, where they can move by LGT into environmental organisms (Chee-Sanford et al., 2001; Nagachinta and Chen, 2008; Schlüter et al., 2008; Gillings et al., 2009a; Pellegrini et al., 2009).

The very use of antibiotics may itself increase the frequency of LGT, and thus the penetration of elements of the mobilome into new bacterial hosts (Beaber et al., 2004; úbeda et al., 2005; Prudhomme et al., 2006). This effect is driven by the bacterial SOS response, which temporarily increases both the basal rate of LGT and of recombination (Tenaillon et al., 2004; Schlacher and Goodman, 2007). There is also evidence that human activities actually select for bacteria with a permanently increased propensity for LGT (Gillings and Stokes, 2012). Consequently, antibiotic pollution creates hotspots for the assembly of complex, mosaic mobile elements from diverse sources, and provides a selective force for their subsequent fixation in diverse lineages (Szczepanowski et al., 2005; Schlüter et al., 2008; Gillings et al., 2009b).

The accumulation of diverse mobile elements within single plasmids or at single loci provides opportunities for complex rearrangements and recombination events that in turn, generate more diversity (Garriss et al., 2009). The emergent properties that arise as mobile elements gain more components means that they can effectively increase their own complexity (Krizova et al., 2011; Toleman and Walsh, 2011). This phenomenon is evident in the increasing complexity and phenotypic plasticity of genomic islands and integrative conjugative elements in emerging nosocomial pathogens such as *Pseudomonas aeruginosa* and *Acinetobacter baumannii *(Fournier et al., 2006; Klockgether et al., 2011).

The selective forces acting upon complex mobile elements are not restricted to antibiotic pressure. Mobile DNAs often carry genes conferring resistance to other selective agents, such as heavy metals, arsenic, and disinfectants. Exposure to any one of these agents selects for lineages containing mobile elements with appropriate resistance genes, and simultaneously fixes all the other genes in physical linkage on that element, including antibiotic resistance determinants. Such co-selection of antibiotic resistance has long been thought to arise as a consequence of pollution with heavy metals (Baker-Austin et al., 2006; Salyers and Shoemaker, 2006; Stepanauskas et al., 2006; Wright et al., 2008; Rosewarne et al., 2010; Drudge et al., 2012). Disinfectant use also results in co-selection of antibiotic resistance (Gaze et al., 2005; Hegstad et al., 2010), and probably had a role in the origin of the class 1 integrons responsible for the widespread dissemination of antibiotic resistance amongst gram negative organisms (Gillings et al., 2008b).

High rates of LGT occur between organisms in similar Phyla, organisms with similar GC content, and organisms that occupy the same environmental niches (Thomas and Nielsen, 2005; Popa and Dagan, 2011). This activity creates lateral exchange communities, where DNA can be exchanged with relative ease (Beiko et al., 2005; Kloesges et al., 2010; Skippington and Ragan, 2011). Shared niches, and in particular, biofilms, promote such exchanges (Sorensen et al., 2005), and both conjugation and natural transformation appear to be important in the process (Kloesges et al., 2010; Domingues et al., 2012). While evolutionary distance and difference in GC content can restrict LGT, there are mechanisms for bypassing these barriers (Popa et al., 2011), and genes can make their way between distantly related organisms via a sequential series of lateral transfers.

Human use of antibiotics is therefore having multiple effects on the mobile components of the bacterial pangenome. One would predict that the size of the mobilome as a proportion of the pangenome is becoming larger, as more genes are recruited onto mobile elements (**Figure 2**). The number of resistome elements now residing on mobile DNA is also increasing, given that plasmids collected before the antibiotic era had no resistance determinants (Datta and Hughes, 1983; Hughes and Datta, 1983), and modern plasmids of similar structure have acquired a wide range of resistance genes dealing with various selective agents in addition to antibiotics. Consequently, there is now a greater overlap between the resistome and mobilome, particularly with respect to genes of concern for human health and welfare (**Figure 2**).

### THE PANGENOME AND GLOBAL MICROBIOME

Human antibiotic use has pervasive effects on both the pangenome and the local composition of the microbiome, largely because antibiotics have non-target effects at gene, cell, and population levels. Exposure of cells to antibiotics induces an SOS response that has widespread effects on bacterial genomes, including raising the rates of mutation, recombination, and LGT (Tenaillon et al., 2004; Aertsen and Michiels, 2006; Schlacher and Goodman, 2007). This increase in basal rates of evolution applies to all the genes and cells in the exposed environment, not just the targets of antibiotic therapy. Consequently, the release of antibiotics into natural environments will affect the diversity of the pangenome and the composition and ecology of the global microbiota (Couce and Blázquez, 2009; Martinez, 2009a; Gillings and Stokes, 2012).

Effects in particular environments have been demonstrated experimentally. Short term antibiotic treatments lead to changes in abundance, richness, and diversity of the human gut microbiota (Dethlefsen et al., 2008), which can remain perturbed for years after treatment (Jakobsson et al., 2010; Sommer and Dantas, 2011). The composition and functional diversity of soil and sediment communities is affected by exposure to antibiotics (Kong et al., 2006; Cordova-Kreylos and Scow, 2007), and persistence of antibiotic residues in sediments or in the water column leads to alteration of microbiota and *in situ* selection for antibiotic resistance (Cabello, 2006; Knapp et al., 2008). While pulses of exposure to antibiotics may only produce transient selection events, alterations to community composition, and the fixation of resistance genes and their mobile vectors may be permanent, with unpredictable consequences for the whole microbiome (Martinez, 2009b; Gillings and Stokes, 2012).

## ANTIBIOTICS AND RESISTANCE GENES AS POLLUTANTS

Antibiotics and their resistance genes originated from natural environments, but human use of antibiotics has perturbed the dynamics of this natural system. The high concentration of antibiotics used prophylactically by humans has two main consequences. Firstly, it leads to antibiotic contamination of human waste streams, and secondly, the selection imposed by antibiotic use has fixed ever more complex genetic elements in commensals and pathogens. These new “xenogenetic” elements are also released via human waste streams. While antibiotics might be treated as simple pollutants, xenogenetic mobile elements are capable of replication, and are thus more akin to invasive species (Gillings and Stokes, 2012).

Between 30 and 90% of the antibiotics given for human or veterinary use are excreted essentially unchanged (Sarmah et al., 2006). These compounds can be both persistent and mobile, and are often not removed during sewage treatment (Watkinson et al., 2007; Le-Minh et al., 2010; Zuccato et al., 2010). Antibiotics are also released in high concentrations from facilities where antibiotics are produced (Li et al., 2009, 2010), and are disseminated during application of manure to agricultural land (Chee-Sanford et al., 2009; Heuer et al., 2011). The consequences of pollution with antibiotics, particularly for aquatic systems, are being actively examined (Taylor et al., 2011; Lupo et al., 2012), and there is a call for development of policies to reduce the release of antibiotics and bacteria via human waste streams (Baquero et al., 2008).

Waste streams that release antibiotics into the environment also disseminate antibiotic resistance genes and mobile DNA elements, which should similarly be regarded as pollutants emanating from human activity (Pruden et al., 2006; Martinez, 2009a; Storteboom et al., 2010; Graham et al., 2011). The abundance of resistance genes in soils has been increasing since the introduction of antibiotics in the 1940s (Knapp et al., 2009), in parallel with the increasing concentrations of more conventional chemical pollutants.

Resistance genes and complex mobile elements are commonly reported from wastewater and sewage treatment plants (Tennstedt et al., 2003; Zhang et al., 2009; Pellegrini et al., 2011). Wastewater is regarded as a hotspot for interactions between mobile elements and for their lateral transfer between pathogens, commensals, and environmental bacteria (Schlüter et al., 2008; Moura et al., 2010). Resistome and mobilome elements are not necessarily removed by water treatment (Graham et al., 2011; Drudge et al., 2012), allowing resistance genes to be used as markers of human influence on aquatic ecosystems (Pei et al., 2006; Pruden et al., 2006; Storteboom et al., 2010).

Domestic and agricultural animals are a source of significant quantities of antibiotics and resistance genes. Animal waste and pig slurry are used to manure soils, with the consequent introduction of resistance genes and resistant bacteria (Binh et al., 2009; Byrne-Bailey et al., 2009; Gaze et al., 2011). The long term fate of antibiotics and resistance genes is difficult to predict without quantitative measurements over appropriate timescales (Chee-Sanford et al., 2009). However, it is clear that antibiotic resistant bacteria will increase in abundance, lateral transfers to soil organisms will occur, and resistance genes will be sequestered by diverse elements of the mobilome (Heuer et al., 2011).

## CONCLUSION

Human use of antibiotics for medicine and agriculture may have consequences beyond their intended applications. Large quantities of antibiotics now emanate from human waste streams, as do the xenogenetic elements fixed in human ecosystems by antibiotic selection. Much more attention needs to be paid to the origins and fates of such pollutants (Allen et al., 2010). The antibiotic revolution may be having effects across the entire microbial biosphere (Martinez, 2009b), changing the basal rate of bacterial evolution, altering the composition of the resistome and mobilome, and promoting lateral transfer of mobile genetic elements (Couce and Blázquez, 2009; Gillings and Stokes, 2012). Antibiotic contamination promotes the fixation and mobilization of resistance genes between environmental and clinical microbiota (Kristiansson et al., 2011), and resistance genes are now widely spread through the biosphere (Martinez, 2009a; Stokes and Gillings, 2011).

Above all, we need to address the antibiotic resistance problem from a broader evolutionary and ecological perspective (Aminov and Mackie, 2007; Baquero et al., 2009; Fajardo et al., 2009). The ability of natural selection to shape species and communities is the same for microorganisms as it is for larger species (Gillings and Stokes, 2012), and the ecological theory of community assembly developed for multicellular organisms can be applied to the microbiome (Costello et al., 2012). The risk associated with the environmental spread of resistance genes with known adverse consequences for human welfare has had little attention, nor has the potential for pollution with antibiotics to widely affect the global microbiome. In comparison, the potential escape of resistance gene markers used in the generation of genetically modified plants has been the subject of considerable research (Martinez, 2012). It is time to pay more attention to the bioactive molecules that humans release into the environment.

## Conflict of Interest Statement

The author declares that the research was conducted in the absence of any commercial or financial relationships that could be construed as a potential conflict of interest.
